# Outcomes 1 year after non-operative management of uncomplicated appendicitis in children: Children with AppendicitiS during the CoronAvirus panDEmic (CASCADE) study

**DOI:** 10.1093/bjsopen/zrad055

**Published:** 2023-06-02

**Authors:** George S Bethell, Clare M Rees, Jonathan Sutcliffe, Nigel J Hall, CASCADE study collaborators, Anna-May Long, Florin Djendov, Victor Emordi, Mark Peter, Sarah Staight, Andrew Jackson, Stewart Cleeve, Arun Kelay, Michael Terry, Christina Major, Oscar Croysdale, Bhavik Patel, Mike Nelson, Eleri Cusick, Hannah Rhodes, Juliette King, Sophie Lewis, Chris Driver, Gillian Winter, Michael Wilson, Rachael Robertson, Duncan Rutherford, Kieran McGivern, Ilhama Jafarli, Selena Curkovic, Raef Jackson, Bhushanrao Jadhav, Maeve Conroy, Thomas Raymond, Vijay Gangalam, Deepak Selvakumar, Khalid Elmalik, Reda Habak, Muslim Abdullah, Mohamed Ahmed Osama, Milan Gopal, Laura Phillips, Khlud Asanai, Hany Gabra, Kamil Naidoo, Noman Zafar, Sophia Lewis, Florence Kashora, Dixa Thakrar, Dean Rex, Annita Budzanowski, Jennifer Binnington, Simon Timbrell, Megan Ridgeway, Shirley Chan, Amani Asour, Adetayo Aderombi, Anna Maria Kocsis, Donald Menzies, Ali Murtada, Corina Dragu, Vincent Quan, Alan Askari, Krashna Patel, Sharukh Zuberi, Saarah Ebrahim, Merrill McHoney, Hetal Patel, Sesi Hotonu, Ashley Meikle, Raj Dass, Andrew Beamish, Rhodri Codd, Rucira Ooi, Alethea Tang, Luke Taylor, Ajay Belgaumkar, Bankole Oyewole, Prabhat Narayan, Marianne Hollyman, Angeliki Kosti, Thomas Badenoch, Asef Rakin, Hamad Khan, Frances Goulder, Katie Siggens, Kizzie Peters, Fiona Kirkham-Wilson, Sophie Bowyer, Enakshee Jamnadass, Paul Froggatt, Karen Lai, Cristina Navarro, Dorinda Chandrabose, Olugbenga Awolaran, Simon Toh, Alex Darbyshire, Ashley Towers, Christine Tan, Joanna Miles, Ingo Jester, Ben Martin, Elmarie Van Der Merwe, Hetal N Patel, Elizabeth Gemmill, Elisa Lenzi, Richard Egan, Keira Soanes, Mark Dilworth, Dimitrios Stamatiou, Alasdair Macmillan, Joshua McIntyre, Danielle Clyde, Majid Rashid, Gandrapu Srinivas, Petros Christopoulos​, Talal Majeed, Katherine Buckley, Darren Smith, Salma Ahmed, Henry Dowson, Gautam Singh, George Kerans, Ashwini Ghorpade, Muhammad Tobbal, Seshu Kumar Bylapudi, Louise Phillips, Kimberley Hallam, Marisa Clemente, Tanzeela Gala, Karol Pal, Lachlan Dick, George Ninkovic-Hall, Emila Paul, Ahmed Abdalla, Theo Pelly, Joe Vance-Daniel, Venkatesh Kanakala, Edward J. Nevins, James Dixon, Michael John, Jude Prince, Kunal Rajput, Rachael Clifford, Siddhant Kumar, Dale Vimalachandran, Georgios Karagiannidis, Fahed Youssef, Suzette Samlalsingh, Chrsitine Ozone, Amina Bouhelal, Siddhartha Handa, Andrew Mitchell, Sathasivam Rajeev, Ellen Ross, Ali Wadah, Tim Bradnock, John Hallett, Felicity Arthur, Shirish Tewari, Vinay Shah, Vivek Gupta, Nick Reay-Jones, Salman Bodla, Nuha Yassin, Harriet Corbett, Sumita Chhabra, Athanasios Tyraskis, Benjamin Allin, Angus Fitchie, Benjamin Samra, Michael Stanton, Dina Fouad, Joshua Brown, Mark Vipond, Harry Dean, Matthew Boal, Oliver Brown, Jonathan Goring, Mahmoud Marei, Christian Verhoef, Jonathan Ducey, Clare Rees, Chipo Mushonga, Dan Frith, Ashok Ram, Tristan Boam, Melissa Gabriel, Ferzine Mohamed, David Williams, Katie Cross, Nadine Dyar, Rick MacMahon, Mohammed Fakhrul-Aldeen, Iain Bain, David Bunting, Graham Branagan, Rachel Carten, Chee Wan Lai, Lydia Longstaff, Charles West, Lucinda Doyle, Anindya Niyogi, Claudia Koh, Michael John, Christian Fox, Brooke Gerrie, Hemanshoo Thakkar, Stavros Loukogeorgakis, Joe Curry, Kate Cross, Jayaram Sivaraj, Sean Marven, Milda Jancauskaite, Helen Please, Wayne Fradley, Fenella Welsh, Maki Jitsumara, Caoimhe Walsh, Sinead Hassett, Ancuta Muntean, Ionica Stoica, Sarah Yassin, Lukas O’Brien, Alan Mortell, Kris Hughes, Maeve Conroy, Khlud Asanai, Suzanne Lawther, David Colvin, Ciaran Durand, Adrian Lim, Mohamed Eltom, Iain Yardley, Kirsty Brennan, Clara Chong, Joshua Pointon, Hasan Mukhtar, Hany Khalil, Stephanie Clark, Mohamad Iskandarani, Ashish Desai, Ben Woodward, Sara Gozzini, Ancuta Muntean, Amulya Saxena, Joshua Cave, Eva Sorensen, Alistair Sharples, Joseph Meilak, Ankur Shah, Sujata Rai, Anang Pangeni, Ashish Kiran Shrestha, Astha Tanwar, Milord Hamal, Marco Youssef, Zaid Al-Hamid, Salma Ahmed, Vasudev Zaver, Jonathan Sutcliffe, Hazem Elfar, Lucy Stephenson, Ed Hannon, Gregory Jones, Jonathan Hodgkinson, Radhika Chadha, James Dale, Timothy Pilpel

**Affiliations:** University Surgical Unit, Faculty of Medicine, University of Southampton, Southampton, UK; Department of Paediatric Surgery, Imperial College Healthcare NHS Trust, London, UK; Paediatric Surgery, Leeds General Infirmary, Leeds, UK; University Surgical Unit, Faculty of Medicine, University of Southampton, Southampton, UK

## Abstract

**Background:**

A major shift in treatment of appendicitis occurred early in the SARS-CoV-2 pandemic with non-operative management used commonly outside research protocols and in units with limited previous experience. This study aims to compare real-world outcomes of surgery *versus* non-operative management of uncomplicated appendicitis in children with 1-year follow-up.

**Method:**

A prospective multicentre observational study of children treated for uncomplicated appendicitis at 74 hospitals in the UK and Ireland from 1 April to 31 July 2020 was performed. Propensity-score matched analysis was conducted using age, sex, C-reactive protein at diagnosis and duration of symptoms as covariates. Primary outcomes were success of non-operative management defined as achieving 1-year follow-up without undergoing appendicectomy due to recurrent appendicitis or ongoing symptoms, and occurrence of any predefined complication (intra-abdominal collection, wound infection, bowel obstruction or reintervention).

**Results:**

Of 1464 children with presumed uncomplicated appendicitis, 1027 (70.2 per cent) underwent surgery and 437 (29.9 per cent) underwent non-operative management. Ninety-four children (21.5 per cent) treated by initial non-operative management required appendicectomy during the index hospital admission while recurrent appendicitis after discharge occurred in 25 (10.4 per cent) children within 1 year. The overall success rate of non-operative management at 1 year was 63.1 per cent (95 per cent c.i. 58.0 to 68.3 per cent). For propensity-score matched analyses, 688 children undergoing surgery and 307 undergoing non-operative management were included. Any predefined complication occurred in 50 (7.3 per cent) children undergoing surgery and in four (1.3 per cent) children undergoing non-operative management (OR 5.9 (95 per cent c.i. 2.1 to 16.6)) in the propensity-score matched cohort. There was no mortality or stoma formation.

**Conclusion:**

Non-operative management is a safe and valid alternative to appendicectomy in children with uncomplicated appendicitis.

## Teaser

This is a prospective multicentre observational study of children treated for uncomplicated appendicitis in the UK and Ireland comparing operative to non-operative management. In 1464 children with presumed uncomplicated appendicitis, operative treatment was associated with greater odds of any complication, intra-abdominal collection and wound infection but lower odds of unplanned general anaesthetic and readmission compared with non-operative management. Non-operative management is a safe and valid alternative to appendicectomy in children with uncomplicated appendicitis.

## Introduction

Non-operative management (NOM) of appendicitis in children has gained increased interest in recent years. Whilst prospective randomized trials are ongoing^1–3^, existing data suggest that NOM is both safe and effective in most children with uncomplicated appendicitis^4,5^. In the UK, NOM of acute appendicitis was largely limited to the setting of a single feasibility RCT before the SARS-CoV-2 pandemic^1,6^. During the pandemic, there was a paradigm shift in practice which has been previously documented and this provides an important opportunity to observe outcomes of NOM in the UK, in a real-world setting^7^. The authors have previously reported initial short-term outcomes of NOM during the pandemic demonstrating that 78 per cent of children with either uncomplicated or complicated appendicitis were discharged home without requirement for appendicectomy^8^. However, knowledge of longer-term outcomes of NOM is critical in informing treatment decisions by surgeons, patients and families.

The aim of this study was to report outcomes at 1 year in a cohort of children treated with either NOM or appendicectomy during the SARS-CoV-2 pandemic. Since the main focus of paediatric RCTs of NOM of appendicitis has been children with uncomplicated appendicitis, and it is for uncomplicated appendicitis that there is greatest interest in understanding the role of NOM as an alternative to appendicectomy, analysis was restricted to patients with uncomplicated appendicitis^1–3,5,9,10^.

## Methods

### Study design and inclusion criteria

Methods have been described in full previously^7^. In brief, this was a prospective multicentre observational cohort study of children aged under 16 years at time of hospital admission with a diagnosis of acute appendicitis. The full study protocol can be found in the *Supplementary material*. All hospitals in the UK and Ireland, including district general hospitals and specialist paediatric surgery centres, were eligible for participation. No changes to diagnostic or treatment pathways were required for inclusion in this study and no specific treatment protocols dictated either appendicectomy or NOM. This study has been reported as per the STROBE statement (*Supplementary material*)^13^.

The diagnosis of appendicitis was based on clinical and/or radiological criteria and only children who were deemed to have uncomplicated appendicitis by the treating surgeon were included in the analysis. Uncomplicated appendicitis was defined as a clinical diagnosis of acute appendicitis by the treating surgeon without suspicion of gangrenous or perforated appendicitis or appendix mass. Children who presented with abdominal pain which was not thought to be appendicitis were excluded, however, those treated for uncomplicated appendicitis initially but then given an alternative diagnosis were included in an intention to treat approach. This study includes all children with an initial admission date between 1 April and 31 July 2020. Follow-up was censored at 1 year postinitial hospital admission date.

### Ethical considerations

This study was registered at each site as a service evaluation, as defined by the health research authority guidance, as this was an observational study only collecting routine anonymized data with no change to clinical care pathways. Given this, individual patient consent was not required.

### Outcomes

Outcomes were taken from a core outcome set of paediatric appendicitis^11^. The primary outcomes were any complication (defined as intra-abdominal collection, wound infection, bowel obstruction and/or reintervention) and success of NOM. Reintervention was defined as a subsequent abdominal surgical or radiological procedure requiring general anaesthesia beyond the initial procedure. Successful NOM was defined as children achieving 1-year follow-up without undergoing appendicectomy due to recurrent appendicitis or ongoing symptoms. Recurrent appendicitis was taken as the diagnosis made by the treating clinician with or without use of imaging. Secondary outcomes were individual complications (including reintervention, intra-abdominal collection, wound infection, bowel obstruction, stoma formation and mortality), readmission, unplanned general anaesthetic and duration of stay. Appendicectomy for recurrent appendicitis in a child initially treated successfully with NOM was not considered as a complication since it is an anticipated event in this treatment pathway.

### Data collection and analysis

Anonymous data were collected by local study teams within each hospital and submitted to the study team monthly with exclusion of duplicates as reported previously^7,8^. Local study teams also returned follow-up data at 1-year after initial hospital admission.

Data are presented as mean (95 per cent c.i.), median (i.q.r. or range if specified) and/or number/total (%) as appropriate. Fisher's exact test or chi-squared test were used for comparison of categorical data and the Mann Whitney–*U* test was used for non-parametric continuous data. A two-tailed *P* value of less than 0.05 was considered as statistically significant. Comparison of outcomes for demographically and clinically matched children treated operatively *versus* NOM was performed using matched propensity-score analysis^12^. Children were matched using age, sex, admission C-reactive protein (CRP) and duration of symptoms using one-to-many matching within a calliper of 0.05, hence excluding those without a matched patient in the other treatment group^12^. These variables were used to allow matching of demographics (age and sex) and disease severity (CRP and duration of symptoms). Following matching, conditional logistic regression or linear regression analysis were undertaken, with results for each outcome reported as odds ratios or days difference with 95 per cent c.i. respectively. Statistical analysis was performed using StataSE v16 (StataCorp LLC, College Station, TX, USA) and commands psmatch2 and stddiff were used for propensity-score analyses. The study was conducted according to the Strengthening the Reporting of Observational studies in Epidemiology (STROBE) guidelines for observational studies^13^.

## Results

### Children included, treatment method and follow-up

A total of 2002 children from 74 hospitals with appendicitis were reported during the study interval of whom 1464 (73.1 per cent) were deemed to have uncomplicated appendicitis and included in the current analysis. Of these, 1027 (70.2 per cent) were treated operatively whilst 437 (29.9 per cent) underwent NOM. Children treated with NOM had a lower CRP and white cell count (WCC) at diagnosis than those treated operatively and were more likely to have had diagnostic ultrasound (*Table 1*). At 1 year after hospital admission data were available for analysis from 1050 of these children (71.7 per cent), including 316 children in the NOM group. Evaluation of differences between cases with and without follow-up data at 1-year analysis (*Table 2*) indicated those with follow-up were more likely to have been treated by a specialist paediatric surgeon, have had a diagnostic ultrasound and, if treated operatively, had undergone laparoscopic rather than open appendicectomy.

**Table 1:** Clinical characteristics and investigation of children treated with operative *versus* non-operative management

	Operative (*n* = 1027)	Non-operative (*n* = 437)	*P*
Age (years), median (i.q.r.)	11 (8–13)	11 (9–13)	0.694
Male	635 (61.8)	264 (60.4)	0.610
**Specialty**			
GS	460 (44.8)	211 (48.3)	0.220
SPS	567 (55.2)	226 (51.7)	
**Laboratory values on admission, median (i.q.r.)**			
WCC—×10^9^/l	14.8 (11.8–17.8)	13.7 (9.9–16.9)	<0.001*
CRP—mg/l	29 (9–69)	21 (5–52)	<0.001*
Ultrasound performed	441 (42.9)	264 (60.4)	<0.001*
CT/MRI performed	27 (2.6)	13 (3.0)	0.710

Values are *n* (%) unless otherwise indicated. *Statistically significant at *P* < 0.05 level. i.q.r., interquartile range; GS, general surgeon; SPS, specialist paediatric surgeon; WCC, white cell count; CRP, C-reactive protein; CT, computed tomography; MRI, magnetic resonance imaging.

**Table 2:** Clinical characteristics and management of children with and without 1 year follow-up

	Follow-up (*n* = 1050)	No follow-up (*n* = 414)	*P*
Age (years), median (i.q.r.)	11 (8–13)	11 (8–13)	0.766
Male	640 (60.1)	259 (62.6)	0.569
**Specialty**			
GS	435 (41.4)	236 (57.0)	<0.001*
SPS	615 (58.6)	178 (43.0)	
**Laboratory values on admission, median (i.q.r.)**			
WCC—×10^9^/l	14.3 (11.0–17.3)	14.9 (11.8–17.8)	0.082
CRP—mg/l	25 (8–62)	29 (7–69)	0.394
Ultrasound performed	556 (53.0)	149 (36.0)	<0.001*
CT/MRI performed	26 (2.5)	14 (3.4)	0.339
Non-operative management	316 (30.1)	121 (29.2)	0.744
Laparoscopic appendicectomy^†^	506 (69.8)	149 (51.2)	<0.001*

Values are *n* (%) unless otherwise indicated. *Statistically significant at *P* < 0.05 level. i.q.r., interquartile range; GS, general surgery; SPS, specialist paediatric surgery; WCC, white cell count; CRP, C-reactive protein; CT, computed tomography; MRI, magnetic resonance imaging. ^†^Initial operative management.

### Operative management

There were 1027 children with suspected uncomplicated appendicitis who had operative management, of whom 655 (63.4 per cent) had a laparoscopic procedure. Intraoperative findings were normal appendix in 57 (5.6 per cent), uncomplicated appendicitis in 683 (67.4 per cent) and complicated appendicitis in 273 (26.9 per cent). Of these, 1-year follow-up data were available for 734 patients (71.5 per cent). Sixty-four (8.7 per cent) children had a related hospital readmission within the first year (median of 1 episode (range 1–4)) with a median time to readmission of 6 days (i.q.r. 3–11, range 1–137). Of these, 29 (4.0 per cent) children had symptoms including abdominal pain but no diagnosed complication.

### NOM

**Fig. 1:**
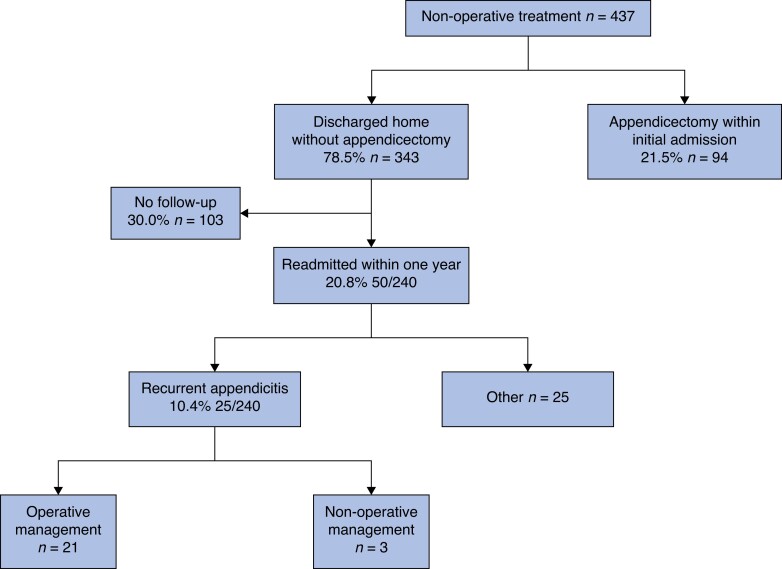
Flow diagram of initial non-operative treatment of appendicitis

There were 437 children treated with NOM (*Fig. 1*). Ninety-four (21.5 per cent) underwent appendicectomy within the index hospital admission with intraoperative findings of normal appendix in six (6.4 per cent) children, uncomplicated appendicitis in 45 (47.9 per cent) and complicated appendicitis in 43 (45.7 per cent). Of the remainder, follow-up data were available for 240 children, with 50 (20.8 per cent) readmitted within 1 year (*Fig. 1*), median time to readmission 48 days (IQR 7.5–144). The median number of readmissions was 1 (range 1–4). Recurrent acute appendicitis was treated in 25/240 (10.4 per cent) children, with 21 undergoing appendicectomy, NOM in 3 children and unspecified/missing management in 1. Other reasons for readmission included abdominal pain and/or fever without a subsequent diagnosis of recurrent appendicitis (*n* = 17), elective appendicectomy for ongoing symptoms but without acute appendicitis (*n* = 4), and planned elective appendicectomy without symptoms (*n* = 4). All appendicectomies after discharge following NOM were performed laparoscopically. In the 94 children who had undergone appendicectomy during initial admission and the 21 who underwent appendicectomy due to recurrent appendicitis, intra-abdominal collection occurred in two children (1.7 per cent), wound infection in one (0.9 per cent), bowel obstruction in one (0.9 per cent) and reintervention was required in one child (0.9 per cent), meaning any complication occurred in four children (3.5 per cent). In total, 123 children underwent appendicectomy within 1 year of index NOM. The overall success rate of NOM, defined as those achieving 1 year follow-up without undergoing appendicectomy due to recurrent appendicitis or ongoing symptoms, was 63.1 per cent (95 per cent c.i. 58.0 to 68.3 per cent (211/334)).

### Comparison of surgical *versus* NOM

Outcomes for children treated operatively and NOM were compared in a matched propensity-score analysis. Matching was possible using the predefined variables in 995/1050 (94.7 per cent) children. Patient characteristics of matched cohorts are shown in *Table S1*. Surgical treatment compared with NOM was associated with greater odds of any complication (OR 5.9 (95 per cent c.i. 2.1 to 16.6)), intra-abdominal collection (OR 5.5 (95 per cent c.i. 1.3 to 23.5)) and wound infection (OR 7.8 (95 per cent c.i. 1.03 to 58.5)) but lower odds of unplanned general anaesthetic (OR 0.05 (95 per cent c.i. 0.03–0.10)) and readmission (OR 0.51 (95 per cent c.i. 0.34 to 0.77)) (*Table 3*). There were no deaths or stoma formation in either group or allergic reactions secondary to antibiotic use.

**Table 3:** Outcomes of children treated with operative *versus* non-operative management–matched propensity-score analysis

	Operative (*n* = 688)	Non-operative (*n* = 307)	Odds ratio or mean difference* (95% c.i.)
**Any complication (reintervention, collection, wound infection, bowel obstruction)**	50 (7.3)	4 (1.3)	5.9 (2.1–16.6)^†^
Reintervention	12 (1.7)	1 (0.3)	5.4 (0.70–42.0)
Intra-abdominal collection	24 (3.5)	2 (0.6)	5.5 (1.3–23.5)^†^
Wound infection	17 (2.5)	1 (0.3)	7.8 (1.03–58.5)^†^
Bowel obstruction	10 (1.5)	1 (0.3)	4.5 (0.57–35.4)
Stoma formation	0 (0)	0 (0)	0.44 (0.01–22.6)
Mortality	0 (0)	0 (0)	0.44 (0.01–22.6)
Readmission	60 (8.7)	48 (15.6)	0.51 (0.34–0.77)^†^
Unplanned general anaesthetic	12 (1.7)	76 (24.7)	0.05 (0.03–0.10)^†^
Initial hospital stay (days), median (i.q.r.)	2 [2–4]	2 [1–3]	0.20* (−0.33 to 0.74)

Values are *n* (%) unless otherwise indicated. ^†^Odds ratios are statistically significant (*P* < 0.05). *Mean difference. i.q.r., interquartile range.

## Discussion

This multicentre prospective cohort study compared outcomes of operative *versus* NOM of uncomplicated appendicitis in children with follow-up to 1 year after initial hospital admission. At 1 year, the success of NOM was over 60 per cent and operative management was associated with significantly increased odds of developing any complication compared with NOM. These data will be useful for counselling children and families when deciding on treatment approach in this common surgical condition.

The success of NOM demonstrated in this study (63.1 per cent) was similar to a large patient preference-controlled study from the USA where the success rate was 67.1 per cent^4^. The exclusion criteria they used meant that only 19.1 per cent of children with appendicitis approached were included in the study. In this observational study, children were included if the treating clinician deemed that the child had uncomplicated appendicitis with no requirement for diagnostic imaging or specific laboratory parameters. This pragmatic approach does mean that some children included had complicated appendicitis and it is possible that the NOM success rate may have been higher if more selective criteria were used. Supporting this, a success rate of 90 per cent at 1 year has been achieved within the confines of an RCT^9^. On the other hand, children without appendicitis may have been included in the NOM group, which might inflate the apparent success rate. Nevertheless, this figure should be generalizable to all types of surgical centres within the UK and Ireland and provides a benchmark for success of NOM. Of note, the given overall success rate of NOM excludes those without 1-year follow-up, which is an unavoidable limitation of this study but results in some uncertainty to the stated outcomes.

As expected in this study of uncomplicated appendicitis, complications were rare in both groups, with reintervention being required in less than 2 per cent of children. Whilst this is reassuring, complications occurred more frequently in the operative group. A recent study exploring patient and parental attitudes of NOM of uncomplicated appendicitis found that a third of participants had a preference for NOM, and avoiding complications of surgery (bleeding and infection) was the second most frequently expressed reason for this preference^14^. With the results of this current direct comparison of complications, there may be greater desire for NOM from children and parents. Readmission and need for unplanned general anaesthetic were seen more frequently in the NOM group, as would be expected, given the recognized risk of recurrence. Parental preference for NOM is reported as up to 63 per cent despite this known risk^15^. Operative complications including intra-abdominal collection and wound infection were seen more commonly with surgical management. These data can be used when discussing management with children and families, and can inform shared decision-making as each individual patient and family may have differing perceptions of the risks and benefits of each approach. Indeed, different preferences and perception of risk have been reported in qualitative work with families who were approached for participation in an RCT of NOM *versus* operative management of appendicitis in children^16^. These comparative data may also be of interest to hospitals and healthcare systems. Studies of adults with appendicitis have revealed cost differences between treatment approaches in favour of NOM^17^. Further work is needed to confirm whether this finding holds true in children^18^.

Outcomes at 1 year following NOM showed no unexpected adverse effects. Those managed by NOM who later required appendicectomy predominantly underwent this via a laparoscopic approach with few postoperative complications and only one of which required reintervention. These findings are comparable to those reported by a similar study undertaken during the COVID-19 pandemic in adults which reported no adverse effects but did report a 1.3 per cent rate of subsequent malignancy in this adult population^17^. Fortunately, malignancy of the appendix is much rarer in children, is more commonly associated with complicated appendicitis and has an excellent prognosis^19,20^. As data on NOM for uncomplicated appendicitis in children evolves, treatment without hospital admission may be considered appropriate, mirroring studies of outpatient antibiotic management of adult appendicitis^21^.

This study is limited by its observational nature, meaning that randomization or treatment protocolization did not occur. Thresholds for converting from NOM to appendicectomy are likely to have varied from surgeon to surgeon. Whilst propensity scoring has been used to provide a matched comparison, the authors cannot adjust for variables that were not measured or subjective variables. The benefit of this approach is that the authors can report on outcomes of a pragmatic study with a relatively large sample size achieved in a short time frame in a real-world setting. Inevitably, data for all cases were not available for inclusion in the 1-year analysis, despite best efforts to obtain them. Patient characteristics were similar for children for whom data were and were not available at 1 year, however, those with follow-up were more likely to be treated by a specialist paediatric surgeon, have had a diagnostic ultrasound and undergone laparoscopic appendicectomy if treated operatively. The authors do not believe this has had a significant impact on the results, which remain generalizable. A final limitation is that although cases were included on the presumption that they had uncomplicated appendicitis, the lack of objective criteria for making this assessment and lack of surrounding evidence base, specifically in children, meant that some in the surgically treated group had more advanced disease and some did not have appendicitis at all. The authors cannot be certain of what proportion of the non-operative treatment group fell into either of these categories and whether complications, such as collections, developed before or after starting treatment. These limitations mean that caution should be exercised when comparing the data reported here with those obtained in prospective RCTs in which diagnosis, case selection and assignment to treatment groups may be more rigorously identified and controlled. Whilst the authors consider it a strength that outcomes are reported to 1 year (one of the longest follow-up intervals for such a large cohort of children), longer follow-up is required particularly in the NOM group to understand whether there is late disease recurrence and the impact of this.

## CASCADE collaborators

Anna-May Long, Florin Djendov, Victor Emordi (Cambridge University Hospitals); Mark Peter, Sarah Staight, Andrew Jackson (Huddersfield Royal Infirmary); Stewart Cleeve, Arun Kelay (Royal London Hospital); Michael Terry, Christina Major, Oscar Croysdale, Bhavik Patel, Mike Nelson (St Mary's Hospital); Eleri Cusick, Hannah Rhodes, Juliette King, Sophie Lewis (Bristol Children's Hospital); Chris Driver, Gillian Winter (Royal Aberdeen Children's Hospital); Michael Wilson, Rachael Robertson, Duncan Rutherford, Kieran McGivern (Forth Valley Royal Hospital); Ilhama Jafarli, Selena Curkovic, Raef Jackson, Bhushanrao Jadhav, Maeve Conroy (University Hospital of Wales); Thomas Raymond, Vijay Gangalam, Deepak Selvakumar (Royal Lancaster Infirmary); Khalid Elmalik, Reda Habak, Muslim Abdullah, Mohamed Ahmed Osama (Leicester Royal Infirmary); Milan Gopal, Laura Phillips, Khlud Asanai, Hany Gabra (Great North Children's Hospital); Kamil Naidoo, Noman Zafar, Sophia Lewis, Florence Kashora, Dixa Thakrar (Northwick Park Hospital); Dean Rex, Annita Budzanowski (St George's Hospital); Jennifer Binnington, Simon Timbrell, Megan Ridgeway (East Lancashire Hospital NHS Trust); Shirley Chan, Amani Asour, Adetayo Aderombi, Anna Maria Kocsis (Medway Maritime Hospital); Donald Menzies, Ali Murtada, Corina Dragu, Vincent Quan (Colchester Hospital); Alan Askari, Krashna Patel, Sharukh Zuberi, Saarah Ebrahim (Luton and Dunstable University Hospital); Merrill McHoney, Hetal Patel, Sesi Hotonu, Ashley Meikle, Raj Dass (Royal Hospital for Children, Edinburgh); Andrew Beamish, Rhodri Codd, Rucira Ooi, Alethea Tang, Luke Taylor (Royal Gwent Hospital, Newport); Ajay Belgaumkar, Bankole Oyewole, Prabhat Narayan (Surrey and Sussex Healthcare NHS Trust); Marianne Hollyman, Angeliki Kosti, Thomas Badenoch, Asef Rakin, Hamad Khan (Musgrove Park Hospital); Frances Goulder, Katie Siggens, Kizzie Peters, Fiona Kirkham-Wilson, Sophie Bowyer, Enakshee Jamnadass (Royal Hampshire County Hospital, Winchester); Paul Froggatt, Karen Lai, Cristina Navarro, Dorinda Chandrabose (Poole Hospital); Olugbenga Awolaran (Royal Alexandra Children's Hospital, Brighton); Simon Toh, Alex Darbyshire, Ashley Towers, Christine Tan, Joanna Miles (Portsmouth Hospital NHS Trust); Ingo Jester, Ben Martin, Elmarie Van Der Merwe, Hetal N. Patel (Birmingham Women's and Children's Hospital); Elizabeth Gemmill, Elisa Lenzi (King's Mill Hospital); Richard Egan, Keira Soanes (Morriston, Swansea); Mark Dilworth, Dimitrios Stamatiou (Birmingham Heartlands Hospital); Alasdair Macmillan, Joshua McIntyre, Danielle Clyde, Majid Rashid (Victoria Hospital Kirkcaldy); Gandrapu Srinivas, Petros Christopoulos​ (Torbay Hospital, Torquay); Talal Majeed, Katherine Buckley, Darren Smith, Salma Ahmed (Wirral University Teaching Hospital NHS Trust); Henry Dowson, Gautam Singh, George Kerans, Ashwini Ghorpade, Muhammad Tobbal (Frimley Park Hospital); Seshu Kumar Bylapudi (Milton Keynes University Hospital); Louise Phillips, Kimberley Hallam, Marisa Clemente, Tanzeela Gala (Glan Clwyd Hospital); Karol Pal, Lachlan Dick (Borders General Hospital, Melrose); George Ninkovic-Hall, Emila Paul, Ahmed Abdalla (Leighton Hospital); Theo Pelly, Joe Vance-Daniel (Kingston Hospital); Venkatesh Kanakala, Edward J. Nevins, James Dixon, Michael John (James Cook University Hospital); Jude Prince, Kunal Rajput, Rachael Clifford, Siddhant Kumar, Dale Vimalachandran (Countess of Chester NHS Foundation Trust); Georgios Karagiannidis, Fahed Youssef (Ipswich Hospital NHS Trust); Suzette Samlalsingh, Chrsitine Ozone, Amina Bouhelal (Queen's Hospital, Barking, Havering and Redbridge University Hospitals NHS Trust); Siddhartha Handa (Furness General Hospital); Andrew Mitchell, Sathasivam Rajeev, Ellen Ross, Ali Wadah (Darlington Memorial Hospital); Tim Bradnock, John Hallett, Felicity Arthur (Royal Hospital for Children, Glasgow); Shirish Tewari, Vinay Shah, Vivek Gupta, Nick Reay-Jones (Lister Hospital); Salman Bodla, Nuha Yassin (New Cross Hospital Wolverhampton); Harriet Corbett, Sumita Chhabra (Alder Hey Children's Hospital); Athanasios Tyraskis, Benjamin Allin, Angus Fitchie, Benjamin Samra (Oxford University Hospital Trust); Michael Stanton, Dina Fouad, Joshua Brown (Southampton Children's Hospital); Mark Vipond, Harry Dean, Matthew Boal, Oliver Brown (Gloucestershire Royal Hospital); Jonathan Goring, Mahmoud Marei, Christian Verhoef, Jonathan Ducey (Royal Manchester Children's Hospital); Clare Rees, Chipo Mushonga, Dan Frith (Imperial College Healthcare NHS Trust); Ashok Ram, Tristan Boam, Melissa Gabriel, Ferzine Mohamed (Norfolk and Norwich University Hospital); David Williams, Katie Cross, Nadine Dyar, Rick MacMahon, Mohammed Fakhrul-Aldeen, Iain Bain, David Bunting (North Devon District Hospital); Graham Branagan, Rachel Carten, Chee Wan Lai, Lydia Longstaff, Charles West, Lucinda Doyle (Salisbury Hospital); Anindya Niyogi, Claudia Koh, Michael John, Christian Fox, Brooke Gerrie (Nottingham Children's Hospital); Hemanshoo Thakkar, Stavros Loukogeorgakis, Joe Curry, Kate Cross, Jayaram Sivaraj (Great Ormond Street Hospital); Sean Marven, Milda Jancauskaite, Helen Please, Wayne Fradley (Sheffield Children's Hospital); Fenella Welsh, Maki Jitsumara, Caoimhe Walsh (Basingstoke Hospital); Sinead Hassett, Ancuta Muntean, Ionica Stoica, Sarah Yassin, Lukas O’Brien, Alan Mortell, Kris Hughes, Maeve Conroy, Khlud Asanai (Children's Health Ireland, Crumlin); Suzanne Lawther, David Colvin, Ciaran Durand, Adrian Lim (Royal Belfast Hospital for Sick Children); Mohamed Eltom (Hull University Teaching Hospital); Iain Yardley, Kirsty Brennan, Clara Chong, Joshua Pointon (Evelina Hospital); Hasan Mukhtar, Hany Khalil, Stephanie Clark, Mohamad Iskandarani (Whittington Health NHS Trust); Ashish Desai, Ben Woodward, Sara Gozzini, Ancuta Muntean (Kings College Hospital); Amulya Saxena, Joshua Cave, Eva Sorensen (Chelsea and Westminster Hospital); Alistair Sharples, Joseph Meilak (University Hospitals of North Midlands); Ankur Shah, Sujata Rai, Anang Pangeni, Ashish Kiran Shrestha, Astha Tanwar, Milord Hamal (William Harvey Hospital); Marco Youssef (Princess of Wales Hospital); Zaid Al-Hamid, Salma Ahmed, Vasudev Zaver (Blackpool Teaching Hospitals NHS Foundation Trust); Jonathan Sutcliffe, Hazem Elfar, Lucy Stephenson, Ed Hannon (Leeds Teaching Hospitals NHS Trust); Gregory Jones, Jonathan Hodgkinson, Radhika Chadha, James Dale, Timothy Pilpel (Royal Berkshire NHS Foundation Trust).

## Supplementary Material

zrad055_Supplementary_Data

## Data Availability

Code used for data analysis and data set are available by reasonable request to the corresponding author.
