# Supplementary material for: Outcomes 1 year after non-operative management of uncomplicated appendicitis in children: Children with AppendicitiS during the CoronAvirus panDEmic (CASCADE) study
Source: BJS Open. 2023 Jun 2;7(3):zrad055. doi: 10.1093/bjsopen/zrad055 (PMC10236944; doi:10.1093/bjsopen/zrad055)
Supplement: zrad055_Supplementary_Data [file zrad055_supplementary_data.docx]

Outcomes 1 year after non-operative management of uncomplicated appendicitis in children: the Children with AppendicitiS during the CoronAvirus panDEmic (CASCADE) study

*George S Bethell^1^, Clare M Rees^2^, Jonathan Sutcliffe^3^, Nigel J Hall^1^ and CASCADE study collaborators.*

Members of the CASCADE study collaborators are listed in manuscript.

1 University of Southampton, University Surgical Unit, Southampton, UK.

2 Imperial College Healthcare NHS Trust, Dept. of Paediatric Surgery, London, UK

3 Leeds General Infirmary, Paediatric Surgery Leeds, UK

**Corresponding author.** Nigel Hall, Associate Professor of Paediatric Surgery, University Surgery Unit, University of Southampton, Southampton, United Kingdom, SO16 6YD. [n.j.hall@soton.ac.uk](mailto:n.j.hall@soton.ac.uk).

**Supplementary Materials - Index**

Study protocol *pag. 2*

STROBE Statement *pag. 3*

Table S1 - Patient characteristics for propensity score matched participants *pag. 5*

|  |  |  |
| --- | --- | --- |
|  |  |  |
|  |  |  |

Children with AppendicitiS during the CoronAvirus panDEmic (CASCADE)

## Introduction

The current coronavirus pandemic is placing NHS services in an unprecedented situation and there will be impact of the delivery of care for non-coronavirus patients. It has been suggested that where alternative non-surgical treatment approaches exist for a given condition, some may be more suitable in these times for a variety of reasons.

The management of children with acute appendicitis is a clinical scenario in which it is recognised there are a number of different treatment options and existing variation in management across the UK. This project aims to understand the impact of the COVID-19 pandemic on the management of children with appendicitis in the UK and to summarise outcomes of this patient population during this time period.

## Methods

This will be a mixed methods study comprising 3 components:

1. A brief survey of consultant surgeons regarding the current and anticipated impact of COVID-19 on the management of children with acute appendicitis to be distributed as soon as possible. All responses will be treated anonymously and an early response summary will be distributed to all participants in the anticipation that the findings may guide or influence practice during the pandemic.
2. A patient level cohort study that will collect data on what treatment was provided to individual cases and what the outcomes were (data will be collected locally and anonymous data forwarded to the coordinating centre on a monthly basis). This will run until the end of the pandemic.
3. At the end of the pandemic a survey of consultant surgeons to understand what happened differently, how effective this was perceived to be, what learning there has been about how we manage appendicitis.

## Data to be collected

A minimum dataset on each case of appendicitis will be recorded using an excel spreadsheet distributed to each participating centre. Each centre will be asked to return the spreadsheet for all cases discharged within a given calendar month.

- The surveys will be administered via REDCap.
- The first survey will be distributed during the week beginning March 30^th^ 2020
- Prospective data collection will start April 1^st^ 2020
- The final survey will be distributed at the end of the pandemic at a timepoint agreed by the study team.

## Centres

All UK centres that treat children with appendicitis are encouraged to participate. Results will be shared with all those who participate as soon as they are available.

## Approvals

Each participating centre will be asked to register this as a service evaluation. The study meets the criteria for a service evaluation according to the HRA guidance.

## Study team

Nigel Hall (Southampton – lead centre), Clare Rees (St Marys Hospital, London), Jonathan Sutcliffe (Leeds), George Bethell (Southampton – Data co-ordinator). All units in the UK that treat children with appendicitis will be encouraged to collaborate with a named consultant and at least one trainee at each centre. If any published article arises from this work then all those who collaborate will be acknowledged under a group authorship model.

## Contact point for queries - [CASCADEstudy2020@gmail.com](mailto:CASCADEstudy2020@gmail.com)

Children with AppendicitiS during the CoronAvirus pandemic (CASCADE) v0.1 March 27^th^ 2020

STROBE Statement—Checklist of items that should be included in reports of ***cohort studies***

|  | Item No | Recommendation | Page No |
| --- | --- | --- | --- |
| **Title and abstract** | 1 | (*a*) Indicate the study’s design with a commonly used term in the title or the abstract | 4 |
|  |  | (*b*) Provide in the abstract an informative and balanced summary of what was done and what was found | 4 |
| Introduction | | | |
| Background/rationale | 2 | Explain the scientific background and rationale for the investigation being reported | 5 |
| Objectives | 3 | State specific objectives, including any prespecified hypotheses | 5 |
| Methods | | | |
| Study design | 4 | Present key elements of study design early in the paper | 5 |
| Setting | 5 | Describe the setting, locations, and relevant dates, including periods of recruitment, exposure, follow-up, and data collection | 5 |
| Participants | 6 | (*a*) Give the eligibility criteria, and the sources and methods of selection of participants. Describe methods of follow-up | 5 |
|  |  | (*b*) For matched studies, give matching criteria and number of exposed and unexposed |  |
| Variables | 7 | Clearly define all outcomes, exposures, predictors, potential confounders, and effect modifiers. Give diagnostic criteria, if applicable | 6 |
| Data sources/ measurement | 8* | For each variable of interest, give sources of data and details of methods of assessment (measurement). Describe comparability of assessment methods if there is more than one group | 6 |
| Bias | 9 | Describe any efforts to address potential sources of bias | 6-7 |
| Study size | 10 | Explain how the study size was arrived at | 5 |
| Quantitative variables | 11 | Explain how quantitative variables were handled in the analyses. If applicable, describe which groupings were chosen and why | 6 |
| Statistical methods | 12 | (*a*) Describe all statistical methods, including those used to control for confounding | 6-7 |
|  |  | (*b*) Describe any methods used to examine subgroups and interactions |  |
|  |  | (*c*) Explain how missing data were addressed |  |
|  |  | (*d*) If applicable, explain how loss to follow-up was addressed |  |
|  |  | (*e*) Describe any sensitivity analyses |  |
| Results | | |  |
| Participants | 13* | (a) Report numbers of individuals at each stage of study—eg numbers potentially eligible, examined for eligibility, confirmed eligible, included in the study, completing follow-up, and analysed | 7-8 |
|  |  | (b) Give reasons for non-participation at each stage |  |
|  |  | (c) Consider use of a flow diagram |  |
| Descriptive data | 14* | (a) Give characteristics of study participants (eg demographic, clinical, social) and information on exposures and potential confounders | 7 |
|  |  | (b) Indicate number of participants with missing data for each variable of interest |  |
|  |  | (c) Summarise follow-up time (eg, average and total amount) |  |
| Outcome data | 15* | Report numbers of outcome events or summary measures over time | 7-8 |

| Main results | 16 | (*a*) Give unadjusted estimates and, if applicable, confounder-adjusted estimates and their precision (eg, 95% confidence interval). Make clear which confounders were adjusted for and why they were included | 7-8 |
| --- | --- | --- | --- |
|  |  | (*b*) Report category boundaries when continuous variables were categorized |  |
|  |  | (*c*) If relevant, consider translating estimates of relative risk into absolute risk for a meaningful time period |  |
| Other analyses | 17 | Report other analyses done—eg analyses of subgroups and interactions, and sensitivity analyses | 8 |
| Discussion | | | |
| Key results | 18 | Summarise key results with reference to study objectives | 8 |
| Limitations | 19 | Discuss limitations of the study, taking into account sources of potential bias or imprecision. Discuss both direction and magnitude of any potential bias | 10 |
| Interpretation | 20 | Give a cautious overall interpretation of results considering objectives, limitations, multiplicity of analyses, results from similar studies, and other relevant evidence | 9-10 |
| Generalisability | 21 | Discuss the generalisability (external validity) of the study results | 10 |
| Other information | | | |
| Funding | 22 | Give the source of funding and the role of the funders for the present study and, if applicable, for the original study on which the present article is based |  |

*Give information separately for exposed and unexposed groups.

**Note:** An Explanation and Elaboration article discusses each checklist item and gives methodological background and published examples of transparent reporting. The STROBE checklist is best used in conjunction with this article (freely available on the Web sites of PLoS Medicine at http://www.plosmedicine.org/, Annals of Internal Medicine at http://www.annals.org/, and Epidemiology at http://www.epidem.com/). Information on the STROBE Initiative is available at http://www.strobe-statement.org.

| Table S1. Patient characteristics for propensity score matched participants | | | | |
| --- | --- | --- | --- | --- |
|  |  | **Operative (n=688)** | **Non operative (n=307)** | **Standardised mean difference** |
| Age, median (IQR) | Years | 11 (8-13) | 11 (8-13) | 0.01 |
| Male |  | 425 (61.8) | 189 (61.6) | 0.01 |
| CRP on admission, median (IQR) | mg/L | 28 (10-62) | 21 (5-52) | 0.10 |
| Symptom duration, median (IQR) | Hours | 26 (24-48) | 24 (24-48) | 0.02 |

Table S1. Patient characteristics for propensity score macthed partcipants. Values are median [inter-quartile range] or n (%). CRP – C-reactive protein; L – litre; mg – milligrams.
